# Robotic-assisted versus manual total hip arthroplasty in obese patients: a retrospective case–control study

**DOI:** 10.1186/s13018-022-03263-6

**Published:** 2022-07-30

**Authors:** Shuai Zhang, Yubo Liu, Minzhi Yang, Mingyang Ma, Zheng Cao, Xiangpeng Kong, Wei Chai

**Affiliations:** 1 Medical School of Chinese PLA, No. 28 Fuxing Road, Haidian District, Beijing, China; 2 Senior Department of Orthopedics, the Forth Medical Center of Chinese PLA General Hospital, No. 51 Fucheng Road, Haidian District, Beijing, 100853 China; 3 National Clinical Research Center for Orthopedics, Sports Medicine and Rehabilitation, No. 28 Fuxing Road, Haidian District, Beijing, China; 4 School of Medicine, Nankai University, No. 94 Weijin Road, Nankai District, Tianjin, China

**Keywords:** Robotics-assisted surgery, Obese, Total hip arthroplasty, Acetabular cup position

## Abstract

**Aims:**

The objective of this study was to evaluate the accuracy of acetabular cup positioning in the obese patients when using robotic-assisted technology during total hip arthroplasty (THA).

**Methods:**

Data were retrospectively collected from patients who underwent primary (THA) with a body mass index (BMI) ≥ 28 kg/m^2^ and ≥ 1 year of follow-up between January 2018 and December 2019. Their demographics, diagnosis, acetabular cup positioning, American Society of Anesthesiologists (ASA) score, Harris Hip Score (HHS), and Forgotten Joint Score (FJS) at the final follow-up were recorded for analysis.

**Results:**

There were no statistically significant differences between the two groups in height, weight, BMI, ASA score, or preoperative Harris Hip Score (HHS). Also, there was no difference in inclination angle between the two groups (R-THA: 41.29° ± 3.04°; manual THA (M-THA): 40.47° ± 5.46°; *P* = 0.312). However, the mean anteversion angle was greater in the R-THA group (20.71° ± 1.98° vs. 19.08° ± 4.04°; *P* < 0.001). Compared to M-THA, R-THA more frequently achieved an acetabular cup angle within 5° of the target (anteversion, 98.1% vs. 78.1% *P* = 0.001; inclination, 88.5% vs. 53.1%, *P* < 0.001). The R-THA group was more advantageous in restoring the hip center of rotation (COR) and leg length difference (LLD). There was no statistical difference in postoperative HHS (*P* = 0.404) or FJS (*P* = 0.497) between the two groups.

**Conclusions:**

Compared to manual technique, robotic-assisted technique provided more precise acetabular cup positioning and better leg length restoration for obese patients. The robotic-assisted technique was more advantageous in recovering the center of rotation position and achieved a higher proportion of the acetabular cup placed in the target safety zone. Further studies are needed to confirm the clinical outcomes of surgeries in obese patients using robotic-assisted technology.

## Introduction

Body mass index (BMI) is defined as the weight in kilograms divided by the square of the height in meters (kg/m2); a BMI ≥ 24 kg/m^2^ is classified as overweight and a BMI ≥ 28 kg/m^2^ as obese, according to Chinese standards [1]. Obesity is a major risk factor for hypertension, diabetes, dyslipidemia, coronary artery disease, myocardial infarction, stroke, breast cancer, and other cancers and has been identified by the World Health Organization as the fifth risk factor affecting health. It is also associated with increased rates of osteoarthritis, particularly in load-bearing joints such as the hip [2].

The increasing prevalence of obesity, combined with the growing demand for THA in the older population, presents a unique set of challenges to the surgeon [3]. Obesity had been shown to negatively affect the outcome of total hip arthroplasty, with a higher incidence of hip dislocation, aseptic loosening, infection, and venous thromboembolism [4–6]. Acetabular cup mispositioning can increase the risk of dislocation, bearing surface wear, and cup instability [7–9]. How to precisely implant cups in THA in obese patients to avoid obesity-related complications and obtain good clinical and radiological results is the focus and difficulty of this procedure.

At our institution, we use robotic-assisted computer navigation technology during THA for more precise positioning of the acetabular cup and better clinical outcomes [10, 11]. This study compared the accuracy of robotic-assisted THA (R-THA) and manual THA (M-THA) for acetabular placement in obese patients, to clarify the value of robotic-assisted techniques for obese patients.

## Materials and methods

This study retrospectively reviewed M-THA and R-THA procedures carried out in our institute between January 2018 and December 2019. Inclusion criteria were: (1): obese patients undergoing primary THA (BMI ≥ 28 kg/m^2^), (2) all surgeries were performed through the posterolateral approach, (3) follow-up time ≥ 1 year. Exclusion criteria were: (1) age less than 18 years old or more than 75 years old, (2) abnormal gait due to neuromuscular insufficiency (including paralysis, rhabdomyolysis, abductor muscle weakness, etc.), (3) combined inflammatory diseases affecting normal activities, cooperation and poor compliance, unable to complete postoperative follow-up assessment, and (4) the patients with incomplete clinical data or nonstandard radiographs. Patients were informed preoperatively that the use of robotic-assisted techniques could result in increased radiation doses, longer operating times, and increased bleeding, costs, and complications. Ultimately, each patient decided whether to undergo robotic-assisted surgery. Patients who met the inclusion criteria were divided into R-THA and M-THA groups according to whether they underwent robotic-assisted technology. All preoperative planning and surgery were performed by one senior surgeon. The MAKO robotic hip system (MAKO Rio Robot; Stryker, USA) was used in our institution, which the system was programmed with THA3.1 for acetabular reaming during bone preparation and cup placement. Institutional review board approval was obtained before starting this study (S2019⁃052⁃01).

### Preoperative planning

Patients scheduled for manual THA had a preoperative plan based on anteroposterior pelvic radiographs using OrthoView 7.0 software (Meridian, UK) to determine cup placement, size, and balance the difference of lower limbs. Patients scheduled for R-THA had a preoperative computed tomography (CT) scan from the iliac wing to the femoral condyle. The CT data were transferred to the MAKO planning module for preoperative planning, with coronal alignment performed based on the anterior superior iliac spine line, anatomical landmarks, and acetabular segmentation modeling. Virtual cup implantation was performed to determine the optimal cup size, angle, and position, which were confirmed by the senior surgeon. The target position of the acetabular cup was 40° of inclination and 20° of anteversion, which could be fine-tuned preoperatively and intraoperatively according to the bone coverage of the acetabular cup.

### Robotic-assisted THA surgical technique

The surgical technique has been described previously [12]. A standardized posterolateral surgical approach was adopted under general anesthesia in both groups. During the procedure, three pins were implanted at the anterior superior iliac spine to fix the pelvic reference frame and fixed adhesive electrodes attached to the patella for intraoperative assessment of leg length. The surgeon began preliminary exposure after the pelvic array had been attached and a locating pin was inserted at the outer edge of the greater trochanter to assess leg length and offset. Subsequently, joint dislocation and femoral neck osteotomy were performed. The acetabulum was registered using a pelvic checkpoint screw inserted outside the acetabulum. The acetabular registration includes 3 acetabular direction determination points, 32 registration points, and 8 confirmation points. The three-dimensional (3D) model based on preoperative CT corresponds to the real anatomical structure of the hip joint. Robotic-assisted acetabular reaming and acetabular cup implantation were performed under 3D real-time navigation according to the preoperative plan. The acetabular screws and liner impacted in place and the femur were prepared manually. Stability of the hip joint was checked by the surgeon through a full range of hip motion and captured the landmarks to measure leg length and offset. Finally, the real femoral stem and head were implanted.

### Manual THA surgical technique

The surgical steps were the same for M-THA, except that the acetabular surgery was performed manually. The smallest reamer was used to determine the acetabular bottom, then the larger reamers in turn to prepare the acetabulum. When the acetabular cup was implanted, we ensured that the cup was implanted at a (20/40) angle by using an antenna. Leg length was assessed intraoperatively by palpating the alignment of the inferior poles of both patellae. Patients in both treatment groups received the Accolade II femoral stem (Stryker, USA) and Trident acetabular shell (Stryker, USA).

### Clinical and radiographic measurements

Patients were followed up routinely at 3, 6, and 12 months postoperatively, and annually thereafter. Anteroposterior pelvic radiographs were taken in the supine position at follow-up, and demographic, radiographic, and surgical data were collected from patients, including gender, age, BMI, diagnosis, acetabular cup positioning, postoperative complications, Harris Hip Score (HHS), and Forgotten Joint Score (FJS). When taking postoperative X-rays, the hips were in 10°–15° of internal rotation and the X-ray beam centered over the pubic symphysis. The longitudinal axis of the body and legs was parallel to the imaging table. The ceramic femoral head was used to calibrate the radiographs to eliminate magnification error. Acetabular cup positioning was measured from supine anteroposterior pelvic radiographs at the 1-year follow-up using OrthoView 7.0 (Meridian), which has shown good accuracy for measuring inclination and anteversion [12–14].

Anteversion was the angle between the short and long axes of the ellipse projected by the cup and was equal to arcsin (short axis/long axis). Inclination of cup was the angle between the cup’s long axis and the trans-teardrop line or the trans-ischial tuberosity line [12, 15–17]. The leg length difference (LLD) was the difference in distance from the line of the bilateral teardrop to the most prominent point of the small trochanter by Ranawat [18]. The horizontal center of rotation (HCOR) was measured as the distance between the center of rotation and a vertical line intersecting the trans-teardrop line, and the vertical center of rotation (VCOR) was measured as the vertical distance between the COR and the trans-teardrop line [19]. The discrepancy between the horizontal and vertical distances of the bilateral COR was taken to evaluate the position of the COR, expressed as HCOR-d and VCOR-d, respectively. Measurements were completed by two experienced physicians, and the interobserver intra-group correlation coefficient was 0.911, indicating good agreement. The final mean value of the two measurements was taken.

### Statistical analysis

Descriptive statistics were presented as means with the standard deviation. The Student’s t test was used for between-group comparisons parametric continuous variables, and categorical variables were analyzed using Chi-squared tests or Fisher’s exact test. All statistical tests were performed at a probability level of 95% (*α* = 0.05). SPSS for Windows (version 25.0; SPSS Inc., Chicago, IL, USA) was used for the analysis.

## Results

### Demographics

A total of 89 patients (116 hips) were included, of whom 41 patients (52 hips) underwent robotic-assisted THA and 48 patients (64 hips) underwent manual THA (Table 1). The mean patient age was lower in the M-THA group (R-THA: 53.38 ± 11.57 vs M-THA: 44.02 ± 12.003 years, *P* < 0.001). There were no statistically significant differences in gender, BMI, diagnosis, ASA, and preoperative HHS between the two groups.

**Table 1:** Demographics

	R-THA	M-THA	*P* value
Patient, *n*	41	48	
Hips, *n*	52	64	
Age (mean ± SD)	53.38 ± 11.57	44.02 ± 12.003	< 0.001
*Sex*			0.202
Male	21	31	
Female	20	17	
BMI (mean ± SD)	30.33 ± 2.387	30.653 ± 2.398	0.47
Preoperative HHS	47.87 ± 13.47	51.25 ± 8.60	0.12
*Diagnosis*			0.295
ONFH, *n*	22	34	
OA, *n*	15	19	
Other, *n*	15	11	
*ASA*			0.646
1	1	1	
2	40	46	
3	0	1	

*BMI* body mass index, *R-THA* robotic-assisted total hip arthroplasty, *M-THA* manual total hip arthroplasty, *HHS* Harris Hip Score, *ONFH* osteonecrosis of the femoral head, *OA* osteoarthrosis, *ASA* American Society of Anesthesiologists

### Acetabular cup positioning

**Fig. 1:**
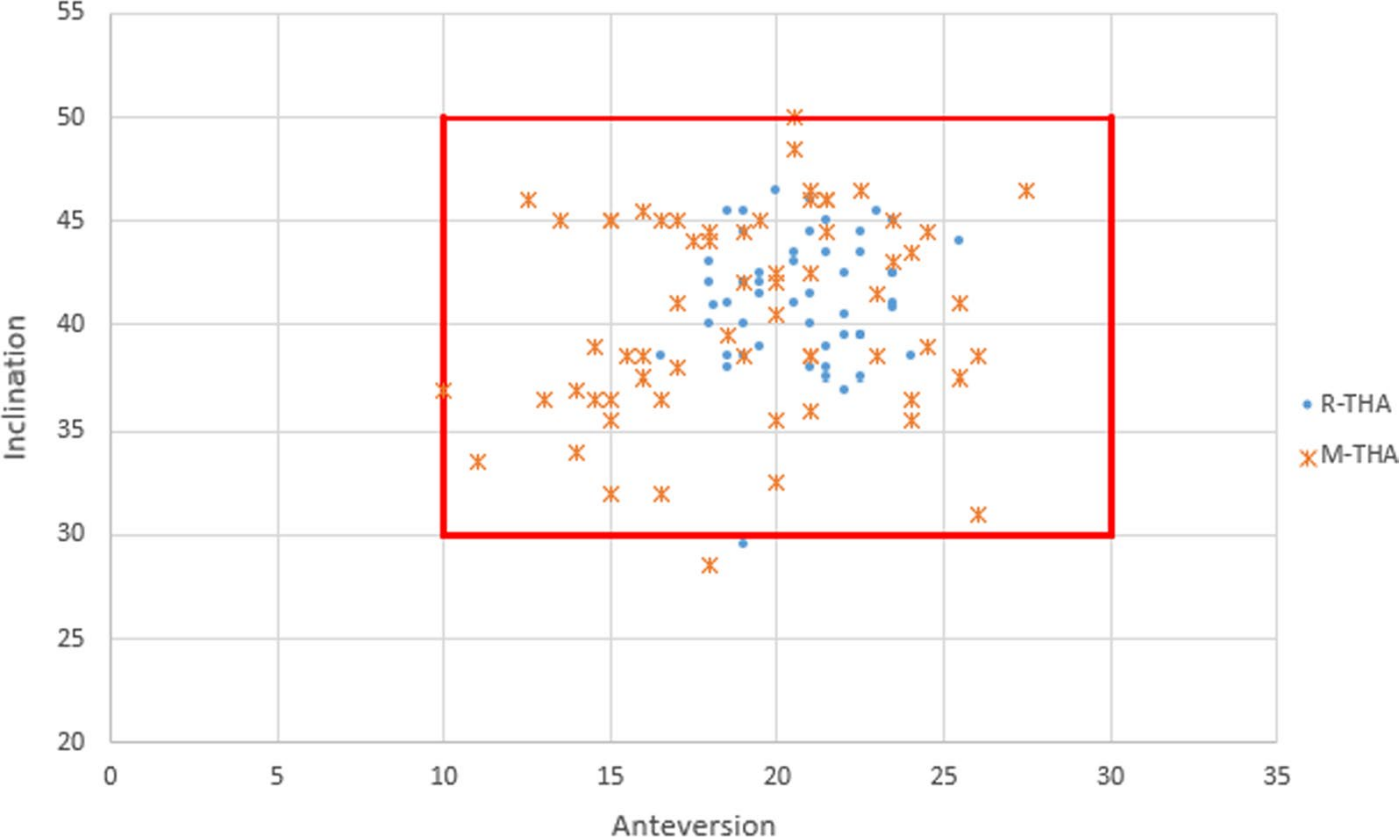
Graphs illustrating the acetabular cup inclination and anteversion. The red box showed the range of the target acetabular component position in anteversion (20° ± 10°) and inclination (40° ± 10°)

**Fig. 2:**
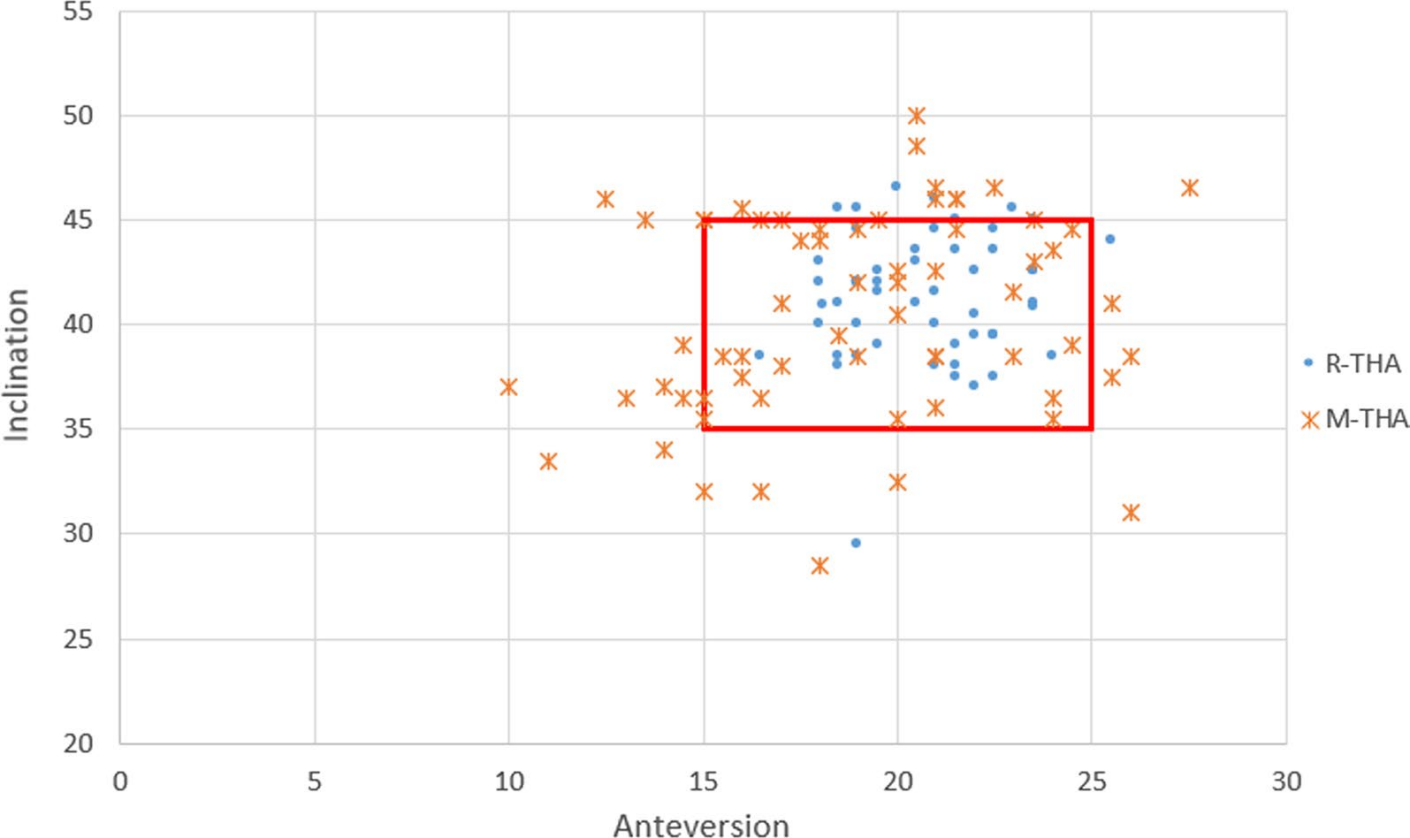
Graphs illustrating the acetabular cup inclination and anteversion. The red box showed the range of the target acetabular component position in anteversion (20° ± 5°) and inclination (40° ± 5°)

The mean inclination in the R-THA and M-THA groups was 41.29° ± 3.04° and 40.47° ± 5.46°, respectively (*P* > 0.05). However, the mean anteversion in the R-THA and M-THA groups was 20.71° ± 1.98° and 19.08° ± 4.04°, respectively, with a statistically significant difference between the two groups (*P* < 0.001) (Table 2). The frequency of an angle within 10° of the target was not different between the R-THA and M-THA groups, but acetabular cup positioning within 5° of the target was significantly (*P* = 0.001) more prevalent in the R-THA group for anteversion (98.1% vs. 78.1%). In the R-THA group, 88.5% of the acetabular cups were within 5° of the target angle with respect to inclination, which was a significantly higher rate compared to the M-THA group (53.1%; *P* < 0.001). The robotic-assisted technique was thus more accurate and consistent with respect to placement of the acetabular cups than manual surgery (Figs. 1, 2) (Table 3).

**Table 2:** Comparison of acetabular cup positioning between R-THA and M-THA

	R-THA	M-THA	*P* value
Inclination (°)	41.29 ± 3.04	40.48 ± 4.81	0.27
Anteversion (°)	20.71 ± 1.98	19.08 ± 4.04	0.005
HCOR-d (mm)	1.6 ± 1.3	2.6 ± 1.7	< 0.001
VCOR-d (mm)	1.9 ± 1	3.1 ± 1.1	< 0.001
LLD (mm)	2.9 ± 1.6	7.3 ± 2.6	< 0.001
LLD ≤ 10(mm)	52/52 (100%)	56/64 (87.5%)	0.008
Postoperative HHS	91.10 ± 4.05	90.39 ± 4.86	0.404
Postoperative FJS	81.40 ± 9.34	80.05 ± 11.65	0.497

*LLD* leg length discrepancy, *HCOR-d* the horizontal center of rotation discrepancy, *VCOR-d* the vertical center of rotation, *HHS* Harris Hip Score, *FJS* Forgotten Joint Score

**Table 3:** Comparison of the rates of anteversion and inclination angles within 5° and 10° of the target acetabular cup angle between R-THA and M-THA

	R-THA	M-THA	*P* value
Anteversion (20° ± 10°)	52/52 (100%)	64/64 (100%)	–
Inclination (40° ± 10°)	51/52 (98.1%)	63/64 (98.4%)	0.882
Anteversion (20° ± 5°)	51/52 (98.1%)	50/64 (78.1%)	0.001
Inclination (40° ± 5°)	46/52 (88.5%)	49/64 (76.6%)	0.098
Anteversion and Inclination within 10°	51/52 (98.1%)	63/64 (98.4%)	0.882
Anteversion and Inclination within 5°	46/52 (88.5%)	36/64 (56%)	< 0.001

The robotic-assisted technique was significantly more accurate than the manual technique in restoring the COR (Table 2) and had a significant advantage in balancing leg length (2.9 ± 1.6 vs. 7.3 ± 2.6 mm, *P* < 0.001), as confirmed by further analysis (Table 2).

### Postoperative function

The HHS and FJS were significantly higher in both groups compared to the preoperative period with no statistical difference between the two groups (HHS: *P* = 0.404; FJS: *P* = 0.497) (Table 2).

### Complications

There were no THA-related complications, signs of cup loosening, and revision during follow-up.

## Discussion

To our knowledge, this is the first case–control study comparing acetabular cup positioning between R-THA and M-THA in obese patients. A previous study showed that higher BMI does not affect the accuracy of cup placement in robot-assisted total hip arthroplasty [10]. However, that study did not compare R-THA to the manual technique, and patient clinical outcomes were not studied. Robotic-assisted THA had been in the clinic for nearly 30 years. Some studies had shown that robotic-assisted THA had advantages in terms of cup position, lower extremity length control, complications, and short-term functional prognosis [12, 15, 20–22]. Another advantage of robotic-assisted technique was the preparation of the surgical plan based on preoperative CT and the use of easily identifiable bone landmarks as reference points during surgery, which can help the surgeon perform procedures in cases where the soft tissue envelope may make accurate implant positioning difficult.

The main difficulties in THA for obese patients include the thick subcutaneous soft tissue, difficulty exposing the acetabulum and femur, and narrow field, which can lead to inadequate intraoperative acetabular bone preparation, poor cup placement, and an increased risk of cup mispositioning [23]. However, the relationship between higher BMI and higher risk of instability or related mechanical complications had not been universally established [4, 24, 25]. Therefore, THA in obese patients presents a formidable challenge to surgeons, and the precise acetabular cup and femoral stem placement were the keys to reduce prosthesis loosening, dislocation, and achieving good outcomes after THA [4, 26]. Accurate acetabular cup positioning and avoidance of cup-related complications remain the primary surgical goals.

We showed that the target acetabular cup positioning could be excellent achieved using both robotic-assisted and manual techniques. However, the standard deviations of both anteversion and inclination were lower in the robot-assisted group. In further analysis, we found that with the target angle of the acetabular cup within 10°, 100% of the anteversion and 98.1% of inclination in both groups were within this range. However, within 5° of the target angles of the acetabular cup, the percentages of the R-THA and M-THA anteversions were 98.1% and 78.1%, respectively, and the inclinations were 88.5% and 53.1%, respectively. This showed that the robotic-assisted technique could be able to achieve the target acetabular cup position (20/40) more precisely and reproducibly than the manual technique in obese patients.

Inaccurate center of rotation (COR) restoration is associated with impingement, reduced hip abductor strength, altered gait kinematics, reduced postoperative hip range of motion, trochanteric pain, instability, and higher rates of wear [19, 27–29]. The horizontal distance of the COR is also considered as the acetabular offset. In addition, a study has shown that the concept of the offset cannot be limited to that of the femoral offset, which acetabular offset widely varies between individuals, and the acetabular floor distance can be up to 13 mm, which should not be ignored by surgeons [30]. Therefore, accurate restoration of the COR is essential for accurate reconstruction of the normal hip anatomy. Our results showed that the robotic-assisted technique was more accurate for restoring the horizontal and vertical distances of centers of rotation with less variation; although the difference between was relatively small, there may be small but important improvements for obese patients.

Leg length discrepancy is a common problem after THA, which is associated with persistent postoperative pain, decreased abductor function, hip instability, impingement, increased surface wear, and early failure, leading to poorer outcomes and patient dissatisfaction, and is a major cause of litigation against orthopedic surgeons [31–33]. Previous studies have shown an obvious advantage of robotic-assisted techniques in restoring leg length discrepancy [15, 22, 34]. This was also demonstrated in our study, as no patients had LLD exceeding 10 mm in the robotic group. In the manual group, there were eight patients with LLD exceeding 10 mm. We analyzed that the first reason could be that obese patients could not achieve the standard lateral position during surgery, resulting in an inability to accurately determine LLD intraoperatively. Second, because obese patients had more soft tissue, which resulted in increased exposure and soft tissue release and the risk of dislocation, therefore, sacrificing leg length was unavoidable in order to achieve satisfactory soft tissue tension and joint stability.

There are several limitations in our study. Firstly, it was a retrospective case–control study, where this design has inherent flaws and biases. Secondly, when comparing the demographics of the two groups, the robotic group was elder (53.38 ± 11.57 vs. 44.02 ± 12.003), which was mainly due to the fact that the MAKO robot system can only use a ceramic-to-polyethylene interface, while relatively younger patients prefer ceramic-to-ceramic prostheses. However, there was no difference in the preoperative functional status of the hip between the two group, and the postoperative follow-up results of both groups were significantly improved and not statistically different compared to the preoperative period, which to a certain extent reflects the advantage of robotic-assisted technology in short-term clinical outcomes. Thirdly, although the accuracy of OrthoView system measurements has been demonstrated, measurement bias cannot be ignored. X-ray-based postoperative measurements are inferior to computed tomography (CT) and magnetic resonance imaging (MRI); however, studies have confirmed that X-rays have the same accuracy as CT [35–37]. Fourthly, due to the relatively small sample size of our study, and the relatively small number of morbidly obese patients in China, no analysis stratified by BMI was conducted. Finally, the follow-up period of patients in this study was greater than 1 year, but it was still a short-term study, in which the long-term clinical outcomes and complications also need to be further followed up.

## Conclusion

Compared to manual technique, robotic-assisted technique provided more precise acetabular cup positioning and better leg length restoration for obese patients. The robotic-assisted technique was more advantageous in recovering the center of rotation position and achieved a higher proportion of the acetabular cup placed in the target safety zone. The postoperative functional scores of the two groups were significantly improved compared with preoperative, and there was no statistical difference. Further studies are needed to confirm the clinical outcomes of surgeries in obese patients using robotic-assisted technology.

## Data Availability

All data generated or analyzed during this study are included in this published article.

## Abbreviations

THA: Total hip arthroplasty
HHS: Harris Hip Score
BMI: Body mass index
SD: Standard deviation
ONFH: Osteonecrosis of the femoral head
OA: Osteoarthrosis
DDH: Developmental dysplasia of hip
CT: Computerized tomography
MRI: Magnetic resonance imaging
R-THA: Robotic-assisted total hip arthroplasty
M-THA: Manual total hip arthroplasty
ASA: American Society of Anesthesiologists
LLD: Leg length discrepancy
COR: Center of rotation
HCOR-d: The horizontal center of rotation discrepancy
VCOR-d: The vertical center of rotation
FJS: Forgotten Joint Score
